# Convenient Heme Nanorod Modified Electrode for Quercetin Sensing by Two Common Electrochemical Methods

**DOI:** 10.3390/mi12121519

**Published:** 2021-12-07

**Authors:** Jin-Guang Liu, Jia-Zheng Wan, Qing-Min Lin, Guo-Cheng Han, Xiao-Zhen Feng, Zhencheng Chen

**Affiliations:** School of Life and Environmental Sciences, Guilin University of Electronic Technology, Guilin 541004, China; liujg1248798982@163.com (J.-G.L.); a1197797116@163.com (J.-Z.W.); lqm442049752@163.com (Q.-M.L.)

**Keywords:** electrochemical sensors, quercetin, detection, heme, modified electrode

## Abstract

Quercetin (Qu) is one of the most abundant flavonoids in the human diet. High concentrations of Qu can easily cause adverse effects and induce inflammation, joint pain and stiffness. In this study, Heme was used as a sensitive element and deposited and formed nanorods on a glassy carbon electrode (GCE) for the detection of Qu. The Heme/GCE sensor was characterized using scanning electron microscopy (SEM), cyclic voltammetry (CV), differential pulse voltammetry (DPV) and electrochemical impedance spectroscopy (EIS) techniques. Under optimized conditions, the developed sensor presented a linear concentration ranging from 0.1 to 700 μmol·L^−1^ according to the CV and DPV methods. The detection limit for the sensor was 0.134 μmol·L^−1^ and its sensitivity was 0.12 μA·μM^−1^·cm^−2^, which were obtained from CV analysis. Through DPV analysis we obtained a detection limit of 0.063 μmol·L^−1^ and a sensitivity of 0.09 μA·μM^−1^·cm^−2^. Finally, this sensor was used to detect the Qu concentration in loquat leaf powder extract, with recovery between 98.55–102.89% and total R.S.D. lower than 3.70%. The constructed electrochemical sensor showed good anti-interference, repeatability and stability, indicating that it is also usable for the rapid detection of Qu in actual samples.

## 1. Introduction

Quercetin (Qu) is a common antioxidant, and is one of the flavonoids that the human body consumes frequently in daily meals [1,2]. Qu mostly exists in the form of glycosides, and it can be obtained through acid hydrolysis and is widely present in the roots, stems and leaves of plants [3]. Qu shows multiple biological activities, such as anti-oxidation, anti-viral, and anti-inflammatory effects, and has demonstrated a great effect in treating prostate cancer and inhibiting the growth of isolated malignant cells [4,5]. Because of the antioxidant properties of Qu, it can remove active oxygen, hydroxyl radicals and superoxide anions very well. However, high concentrations of Qu tend to cause reverse effects and induce inflammation, joint pain and stiffness, and can also cause abnormal changes in hormone levels in the body [6,7,8,9]. Therefore, the determination of Qu content is of great significance.

There are currently many methods used to detect Qu in clinical applications, including the fluorescence method [10], spectrophotometry method [11], the capillary electrophoresis (CE) method [12] and the high-performance liquid chromatography (HPLC) method [13,14]. However, these methods have inherent problems, such as time-consuming processes, high cost, and laborious processes. Nowadays, the methods for Qu detection have been continuously innovated and improved. Electrochemical analysis is an especially excellent method because it is real-time, rapid, sensitive and easy to miniaturize [15,16]. Previous studies have shown that electrochemical detection is very suitable for the detection of Qu [17,18]. For example, Arvand et al. [19] successfully prepared an amperometric determination sensor and used magnetic Core/Shell Fe_3_O_4_@ZnO nanoparticles modified on GCE to detect Qu by means of cyclic voltammetry (CV), with a detection limit of 0.16 μmol·L^−1^. Ma et al. [20] used three-dimensional reduced graphene oxide aerogel modified on electrodes to construct a sensor to realize the detection of Qu by means of differential pulse voltammetry (DPV), with a detection limit of 0.065 μmol·L^−1^. All the mentioned electrochemical sensors for the detection of Qu were attributed to the electrochemical signal amplification function used by the prepared modifier. Therefore, it is important to find the right modifier. Heme plays an important role in the construction of many sensors because that Heme can amplify the signal of the detected substances [21,22,23,24,25].

Herein, an electrochemical sensor with high sensitivity for signal amplification was developed to detect Qu by means of a Heme nanorod with great catalytic effect. After a series of characterizations to analyze the construction of the sensor, the as-prepared Heme/GCE electrochemical sensor was found to be usable for the rapid detection of Qu in loquat leaf samples through the DPV technique.

## 2. Materials and Methods

### 2.1. Chemicals and Reagents

Quercetin (Qu) and Heme were purchased from Aladdin Biochemical Technology Co. Ltd. (Shanghai, China), β-cyclodextrin (β-CD) was provided by Macleans Biochemical Technology Co. Ltd. (Shanghai, China), dopamine hydrochloride (DA) was obtained from Maoerwo Bio-Pharmaceutical Co. Ltd. (Hubei, China), ferrocenylglutathione (Fc-ECG) was provided by Chang Xi Biotechnology Co. Ltd. (Shanghai, China), and loquat leaf powder was purchased from market, which was directly diluted with phosphate-buffered saline (PBS) buffer for DPV experiments. All other reagents were of analytical grade and were used without further purification. All aqueous solutions were prepared with Milli-Q water.

### 2.2. Instrumentation

A three-electrode system was used to conduct electrochemical analyses, in which the counter, reference and working electrodes were a Pt electrode, an Ag/AgCl, and the GCE (φ 3 mm), respectively. The obtained data were acquired using a CHI660E electrochemical workstation (Shanghai Chenhua Instrument Co., Ltd., Shanghai, China), which was attached to the three-electrode system for testing. All screen-printed electrodes (SPEs) were provided by Nanjing Yunyou Biotechnology Co.Ltd. (Nanjing, China). Under optimized conditions, DPV experiments were applied at a scanning interval from −0.20 to 0.60 V. CV experiments were performed with a scanning interval from −0.40 to 1.20 V at the scanning rate of 0.1 V·s^−1^. EIS analyses were carried out to characterize the electron transfer ability of sensors. EIS measurement was performed in 5.0 mmol·L^−1^ of K_3_[Fe(CN)_6_]/K_4_[Fe(CN)_6_], which was prepared with deionized water in a frequency range from 10,000 to 0.1 Hz, an amplitude of 5 mV and an initial voltage of 0.16 V. All the test EIS data were fitted using ZsimpWin software. A field emission scanning electron microscope (Hitachi, Japan) was used to characterize the surface morphology for electrochemical sensors with the working distance of 6000 μm at 3 kV and 10,100 nA. In addition, it is necessary to explain the calculation method used for the detection limit and sensitivity. The calculation method for the detection limit was 3S/K and the sensitivity calculation method was K/A. S is the standard deviation of multiple tests of blank samples, K is the slope of the linear equation and A is the area of the working electrode.

### 2.3. Fabrication of Sensor and Qu Sensing

**Scheme 1:**
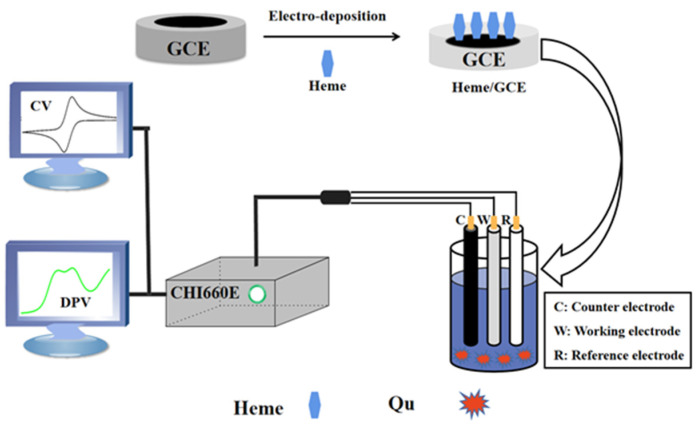
The fabrication of the Heme/GCE sensor for the detection of Qu by means of the CV and DPV techniques.

GCE was polished with aluminum oxide powder and cleaned with ethnaol and Milli-Q water. To prepare the Heme/GCE sensor, 1 mL 250 μmol·L^−^^1^ Heme solution dissolved in 3 mL PBS (pH 5.7) was deposited for 120 s using the *i-t* method at a constant potential of 0.10 V. The preparation processes of the DA/GCE sensor, β-CD/GCE sensor and Fc-ECG/GCE sensor were exactly same as that of the Heme/GCE sensor for Qu sensing, using two straightforward electrochemical methods including CV and DPV, as illustrated in Scheme 1.

### 2.4. Preparation of Actual Sample

Commercially available loquat leaf powder of 2.040 g was dissolved in 20 mL 70% ethanol. After a 30 min ultrasound, it was soaked for 48 h and then sonicated for 30 min again. The filter residue pumped from the mixture was washed three times with an appropriate amount of distilled water. Twenty milliliters of dilute hydrochloric acid (pH 1.0) were added for acid hydrolysis and sodium hydroxide (pH 10.0) was added to adjust the pH value of the sample to 5.7. The processed sample solution was mixed with PBS (pH 5.7) in a ratio of 1:1, and an appropriate amount of NaCl was added as an electrolyte. The obtained sample solution was used for further investigation.

## 3. Results and Discussion

### 3.1. Electrochemical Properties of Different Sensors

First, the DPV and EIS techniques were used to conduct electrochemical analyses of the sensor construction process to further demonstrate that β-CD, DA, Heme and Fc-ECG were successfully fixed on the working electrode surface, respectively, indicating the electrochemical properties of different sensors at the same time. The experimental results are shown in Figure S1.

Figure S1A shows the DPV graphs of different working electrodes scanned in PBS (pH 7.0). Curve a is bare GCE, curve b is β-CD/GCE, curve c is DA/GCE, curve d is Fc-ECG/GCE and curve e is Heme/GCE. Compared with naked GCE (curve a), β-CD/GCE, DA/GCE and Heme/GCE, Fc-ECG/GCE had characteristic potentials around −0.10 V, 0.20 V, −0.20 V and 0.40 V. The peak potentials of β-CD, DA, Heme and Fc-ECG were −0.10 V, 0.20 V, −0.20 V and 0.40 V, respectively, with peak current values around 3.80, 4.30, 1.90 and 1.0 μA, respectively. These results proved that β-CD, DA, Heme and Fc-ECG were successfully modified on GCE.

The EIS method is a very useful technique for detecting the properties of the electrode interface and it can be used to characterize the entire process of sensor construction. Figure S1B demonstrates the EIS curves of different constructed electrodes and the insert presents an enlarged view. The EIS curve of naked GCE can be seen as a half arc. After β-CD, DA, Heme and Fc-ECG were deposited on the surface of the electrode, the curve changed. Accordingly, the impedance changes confirm that the modifiers β-CD, DA, Heme and Fc-ECG were successfully modified on the electrode surface, which is consistent with the DPV results and the fitting results (Figure S1C).

Then, the CV and DPV methods were used to study the electrochemical responses of Qu sensing using different working electrodes, and the substance with the strongest electrochemical catalysis for Qu was selected as the modifier to detect Qu. Figure S2 shows the electrochemical behavior of 10 μmol·L^−1^ Qu on different working electrodes.

It can be seen in Figure S2A that a couple of redox peaks of Qu, as detected by CV method, were found around 0.20 V. Obviously, the Heme-modified electrode had the highest current value, and was more conducive to the electrocatalysis of Qu. As illustrated in Figure S2B, the peak potential of Qu was concentrated at about 0.20 V according to the DPV method, and the peak current of Qu on the naked GCE electrode was 0.63 μA, whereas the peak currents detected using β-CD/GCE, DA/GCE, Fc-ECG/GCE and Heme/GCE were 1.14, 1.40, 1.51 and 2.27 μA, respectively. This suggests that the β-CD/GCE, DA/GCE, Fc-ECG/GCE and Heme/GCE electrodes could enhance the current response of Qu by 1.81, 2.22, 2.40 and 3.63 times compared with naked GCE, respectively. It can be seen in Figure S2C,D that the dynamic process of Qu is in accordance with the Randles–Sevcik equation. The effective surface area of Heme/GCE was calculated as 0.12 cm^2^ using the Randles–Sevcik equation, and this showed an increase when compared to the naked GCE (0.07 cm^2^). Herein, Heme displayed a signal amplification effect. The reason for this is to increase the surface area of the electrode, and a more critical factor is the specific catalytic effect of Heme on Qu. Therefore, Heme was selected as the modifier for Qu detection.

### 3.2. SEM Characterization of Heme/GCE

**Figure 1:**
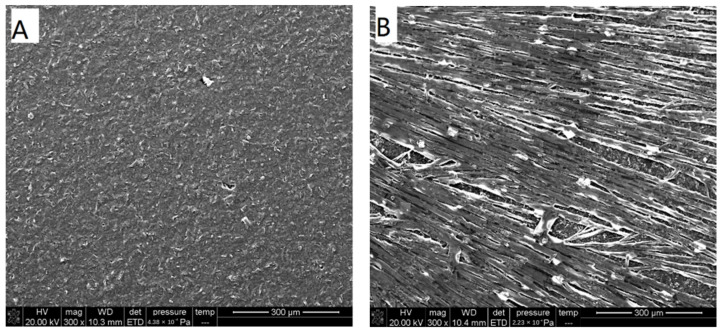
SEM images of different electrodes ((**A**) naked SPE; (**B**) Heme/SPE).

Secondly, in order to observe whether Heme had been modified on the SPE, scanning electron microscopy (SEM) was carried out to observe its surface topography. Here, screen-printed electrodes (SPEs) were used to replace GCE. Figure 1 shows SEM images of two electrodes.

It can be seen in Figure 1A that many pure carbon particles were distributed on SPE. There are bright spots and thin rods visible in Figure 1B, which reveals that Heme was successfully modified on SPE. Heme nanorods with coincident orientation can accelerate electron transfer, which leads to higher catalysis performance.

### 3.3. Optimization of Experimental Conditions

Thirdly, different concentrations of modifier Heme, deposition time, pH value and temperature are also related to vast differences in the electrocatalytic effects of Qu. Figure S3A,B show the CV and DPV graphs of different concentrations of Heme modified on GCE for Qu detection, respectively. Figure S3C,D show the CV and DPV graphs of different deposition times of Heme on the GCE surface. Figure S3E,F display the current response for Qu detected in PBS of different pH values. Figure S3G,H show the relationship between peak potential and pH value according to the CV and DPV methods, Figure S3I,J show the CV and DPV graphs for Qu detection at different temperatures, respectively.

As shown in Figure S3A,B, the peak current value became higher as the Heme concentration increased. However, it did not change much after the concentration of Heme rose from 250 to 300 μmol·L^−1^. Considering the experimental results above, 250 μmol·L^−1^ Heme was selected as the modification value for the subsequent experiment.

The amount of modifiers enriched on the electrode surface also affects the current value of Qu to a large extent. It can be seen in Figure S3C,D that when the deposition time was 120 s, the peak current value reached the maximum. In the CV diagram the maximum current is 1.90 μA and in the DPV diagram it is 2.40 μA. After the deposition time exceeded 120 s, the peak current value decreased, which may be attributed to the effect of excessive Heme deposition. Compared to the various deposition times, 120 s was the best deposition time for subsequent experimental research.

Generally speaking, the pH of the solution can affect the activity of Qu. It can be seen in Figure S3E,F that as the buffer with Qu was more alkaline, the peak potential shifted further to the left, whereas when the buffer with Qu was more acidic, the peak potential shifted further to the right. The current value of Qu was the highest when the pH was 5.7; therefore, the buffer with Qu (pH 5.7) was selected for the later experiment. Figure S3G shows the corresponding plot of oxidation peak potential and pH. The peak potential was linearly shifted to a more positive value as pH varied from 5.7 to 8. The linear regression equation is E_p_ = −0.068 pH + 0.685, with a correlation coefficient of R^2^ = 0.997. The calculated slope value of −68 mV/pH is close to the theoretical Nernstian value (−59 mV/pH), thus suggesting that an equal number of two protons and two electrons is involved in the oxidation of Qu on the electrode surface [26]. Figure S3H, which presents DPV analysis, also illustrates similar results.

Temperature is an important factor affecting the activity of the substance itself, so an appropriate temperature was chosen for subsequent detection. As can be seen in Figure S3I,J, the response current of Qu increased slightly with the increase of temperature (at 4, 15, 25, 30 and 37 °C), indicating that temperature had a poor effect on the activity of Qu. Considering the relative temperature control of the environment in difficult cases, room temperature (25 °C) was chosen to carry out further experiments.

### 3.4. Qu Analysis

**Figure 2:**
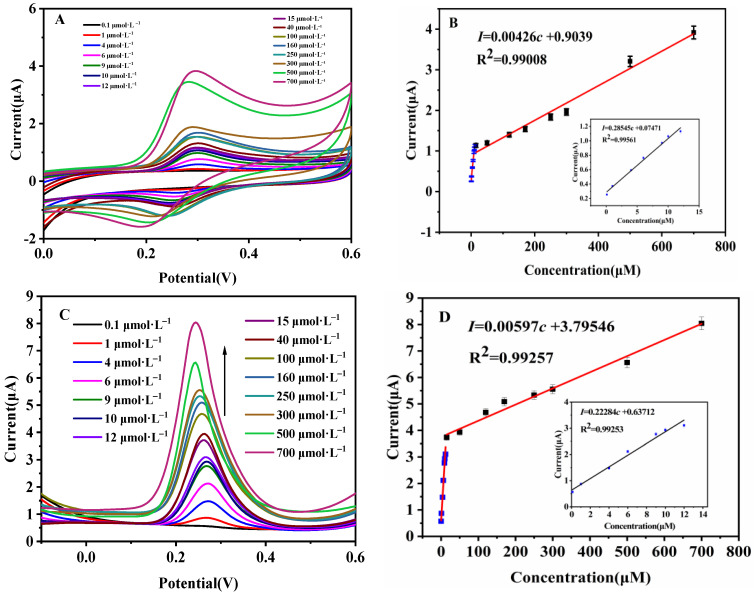
(**A**) CV graph of Qu at different concentrations, (**B**) the linear equation graph corresponding to graph (**A**), (**C**) DPV graph of Qu at different concentrations, (**D**) corresponding linear equation diagram corresponding to graph (**C**) (the illustrations of (**B**,**D**) are low-concentration linear equation diagrams).

Lastly, under the optimal conditions, the detection for different concentrations of Qu was carried out by Heme/GCE via CV and DPV. With the results shown in Figure 2, Figure 2A,C are the CV graph and the DPV graph of different Qu concentrations; Figure 2B,D display the working curve diagrams corresponding to Figure 2A,C, respectively; and the inserts are low-concentration working curve diagrams.

As shown in Figure 2A, the CV method was used to study the electrochemical signal of different concentrations of Qu (0.1, 1, 4, 6, 9, 10, 12, 15, 40, 100, 160, 250, 300, 500 and 700 μmol·L^−1^) detected by means of Heme/GCE. The corresponding linear equation is shown in Figure 2B. When the concentration range is 15–700 μmol·L^−1^, a good linear relationship *I*(μA) = 0.9039 + 0.00426*c* (μmol·L^−1^) and R^2^ = 0.99008 are obtained. The insert of Figure 2B shows the linear realationship *I*(μA) = 0.07471 + 0.28545*c* (μmol·L^−1^), with a correlation coefficient (R^2^) of 0.99561 in the range of 0.1–12 μmol·L^−1^. The detection limit was calculated as 0.134 μmol·L^−1^ and its sensitivity was 0.12 μA·μM^−1^·cm^−2^.

With DPV applied, as illustrated in Figure 2C, a good linear relationship between the concentration of Qu and the current response was also obtained. As shown in the insert of Figure 2D, the linear equation is *I*(μA) = 0.63712 + 0.22284*c* (μmol·L^−1^) with R^2^ = 0.99253 when the concentration ranged from 0.1–12 μmol·L^−1^. As shown in Figure 2D, the linear equation is *I*(μA) = 3.79564 + 0.00597*c* (μmol·L^−1^) with R^2^ = 0.99257 in the range from 15 to 700 μmol·L^−1^. The detection limit of the constructed sensor was 0.063 μmol·L^−1^, and its sensitivity was 0.09 μA·μM^−1^·cm^−2^.

Table S1 shows a comparison of the Qu detection results of different methods. Compared with that of other methods, the linear range of the method outlined in work is relatively wide and the detection limit is relatively good. In Kathiravan’s research [27], a multilayer sensor (MIP/MIL-101(Cr)/MoS_2_/GCE) was successfully prepared for the detection of Qu, whereas the sensor in this work only modified one layer, but they have the same linear range (0.1–700 μmol·L^−1^), indicating that the use of the Heme/GCE sensor represents a more convenient alternative.

### 3.5. Heme/GCE Sensor Anti-Interferences, Reproductivity and Stability Study

**Figure 3:**
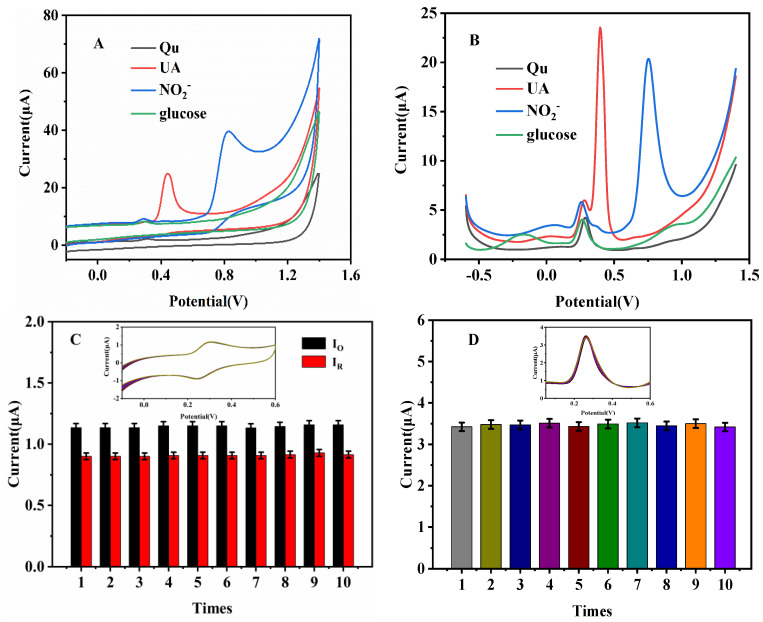

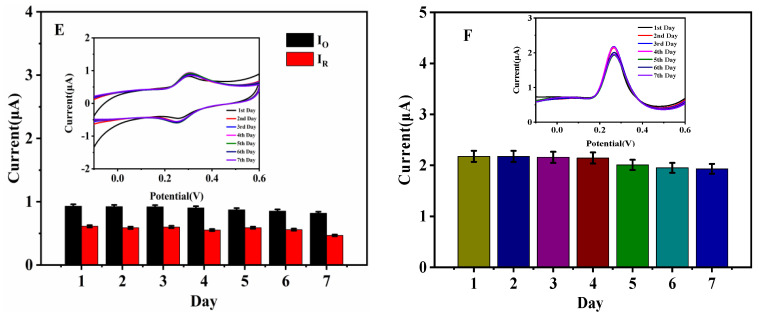
Anti-interference studies of Heme/GCE. (**A**,**B**) are the CV and DPV diagrams of the mixed solution of 10 μmol·L^−1^ Qu and interference substances. Reproductivity and stability studies of Heme/GCE. (**C**,**D**) are the CV and DPV graphs of scanning the same Qu solution using the 10 same electrodes; (**E**,**F**) are the CV and DPV graphs of scanning for seven consecutive days (the inset is the peak current value histogram).

The anti-interference, reproductivity and stability of the constructed electrochemical sensor were investigated as well, and the results are shown in Figure 3. Figure 3A,B are graphs of the CV and DPV response with interference added. Figure 3C,D are histograms of 10 measurements for Qu using the same ten Heme/GCE sensors according to CV and DPV, respectively. Figure 3E,F present the CV and DPV responses of continuous Qu detection using the Heme/GCE sensor over seven consecutive days.

The anti-interference of the constructed electrochemical sensor was investigated. We selected 10 μmol·L^−1^ Qu and 1 mmol·L^−1^ NO_2_^−^, uric acid (UA) and glucose (with a 1:100 concentration) for investigation using the CV and DPV methods. It can be seen in Figure 3A, the oxidation peak potentials of three interferences can be separated from the oxidation peak of Qu (0.30 V). The peak potentials of UA and NO_2_^−^ were 0.45 V and 0.83 V, respectively. Although UA had a peak potential near 0.4 V, its peak current was larger, so it could be distinguished from Qu. The concentration of all interferences were 100 times that of Qu; in this case, the Qu signal could be detected, which was enough to show that the sensor has good selectivity and anti-interference. As depicted in Figure 3B, when DPV was applied, the peak of Qu, UA, NO_2_^−^ and glucose were at 0.25, 0.40, 0.80 and 1.00 V, respectively. After these interference substances were added to Qu samples, the current value did not change much. This reveals that the prepared Heme/GCE sensor has good anti-interference performance.

To verify the reproductivity of the constructed electrochemical sensor, ten Heme/GCE sensors were carried out for the detection of Qu. The results were illustrated in Figure 3C,D (the insets depict bar graphs of the current value). The average R.S.D. of the measured values of the Qu oxidation peak and the reduction peak obtained using the CV method were 0.87% and 0.97%, respectively. The average R.S.D. of the peak current values detected via DPV was 1.10%. These results show that the constructed electrochemical sensor has good reproductivity.

The stability of the prepared sensor was also investigated by testing 10 μmol·L^−1^ Qu samples for seven consecutive days. The average R.S.D. of the oxidation peak and the reduction peak were 4.77% and 8.60%, respectively, and the R.S.D. of the DPV peak value was 5.27%, which showed a satisfactory stability.

### 3.6. Sample Analysis

By comparing the sensitivity, the detection limit and wave of CV and DPV methods, it is concluded that the DPV method for Qu detection is better than the CV method; hence, DPV method was subsequently used to detect actual samples.

The DPV method was used to test the prepared actual samples above, with 0, 50, 250, 500 μmol·L^−1^ Qu added three times using the standard addition method. The data results are shown in Table 1, the average current value was 2.14 μA when detecting real samples without any Qu added and the actual sample detection curve is shown in Figure S4. After it was calculated using the linear equation. The concentration of Qu in the loquat leaf powder extract sample was 8.82 μmol·L^−1^ and the R.S.D. was 3.70%. The average recovery of spiked samples was from 98.55% to 102.89%. This indicated that the as-prepared sensors possess the potential for practical application in real samples. In addition, the concentration obtained by detecting the actual sample using the ultraviolet (UV-Vis) method (Appendix A) was 12.36 μmol·L^−1^; the test results are shown in Figure S5. Since the loquat leaves were not strictly pre-treated, the other components in the loquat leaves interfered with UV-Vis, which caused the absorption peak intensity at 260 nm and 380 nm to be greatly reduced or even to disappear. Another reason may be that Qu is a diffusion control process in the solution, and fewer Qu molecules slowly move to the surface of the electrode and lead to a small current response. Therefore, this led to the differences between the two methods, which is inevitable within an acceptable rage.

## 4. Conclusions

In this study, we prepared a very simple nanorod Heme/GCE sensor for Qu detection by means of the CV and DPV techniques. The results show that the electrocatalytic performance of Heme/GCE has an excellent effect on Qu. Under optimal conditions, two good linear relationships with a wide range of 0.1–700 μmol·L^−1^ were obtained by means of the CV and DPV methods, the detection limits for the sensor were 0.134 and 0.063 μmol·L^−1^, and the sensitivity values were 0.12 and 0.09 μA·μM^−1^·cm^−2^, respectively. The sensor showed great anti-interference properties, reproductivity and stability. The R.S.D. of the detection of the loquat leaf powder extract was 3.70%. The average recovery ranged from 98.55% to 102.89%, according to the standard addition method. These results prove that the Heme/GCE sensor provides an effective method in practical applications.

## Figures and Tables

**Table 1:** Results of Qu detection in loquat leaf powder extract samples (n = 3).

Added Concentration of Qu (μM)	Current I (μA)	Average Found (μM)	Recovery (%)
0	2.14 ± 0.05	8.82 ± 0.26	-
50	3.56 ± 0.13	45.42 ± 15.67	98.55%
250	4.53 ± 0.22	240.29 ± 35.94	99.53%
500	5.80 ± 0.02	496.47 ± 6.63	102.89%

## Data Availability

Data are contained within the article.

## Supplementary Materials

The following are available online at https://www.mdpi.com/article/10.3390/mi12121519/s1, Figure S1: DPV graphs(A) of different working electrodes in PBS buffer (pH 7.0) and EIS diagrams(B) of different working electrodes in 5 mmol·L^−1^ K_3_[Fe(CN)_6_]/K_4_[Fe(CN) _6_] solution; EIS fitting diagrams (C), Figure S2: CV (A)and DPV(B) diagrams of 10 μmol·L^−1^ Qu on different working electrodes; (C) Cyclic voltammograms of Qu at Heme/GCE with different scan rate (10–200 mV/s); (D) The plots of anodic peak currents (*I*p) and Qu vs. ν^0.5^, Figure S3: 10 μmol·L^−1^ Qu on working electrodes modified with different Heme concentrations (A) CV graph and (B) DPV graph; (C) CV graph and (D) DPV graph of the 10 μmol·L^−1^ Qu under different Heme deposition time; (E) CV graph and (F) DPV graph of the 10 μmol·L^−1^ Qu detected in PBS buffer with different pH; (G) CV graph and (H) DPV graph of the 10 μmol·L^−1^ Qu at different temperatures; (I) CV graph and (J) DPV graph of the 10 μmol·L^−1^ Qu at different temperatures, Figure S4: The DPV diagram of the actual sample tested three times in a row, Figure S5: (A) UV-Vis spectra of different concentration of standard Qu solution, (B) the linear fitting curve of UV detection, and (C) UV-Vis spectra of actual samples were detected by three times, Table S1: Comparison with other Qu detection methods.

## Appendix A

All of the ultraviolet (UV-Vis) visible absorption spectra were measured on the Hitachi UH5300 from 190 to 900 nm.

## References

[1] Zaplatic E., Bule M., Shah S.Z.A., Uddin M.S., Niaz K. (2019). Molecular mechanisms underlying protective role of quercetin in attenuating Alzheimer’s disease. Life Sci..

[2] Patel R.V., Mistry B.M., Shinde S.K., Syed R., Singh V., Shin H.S. (2018). Therapeutic potential of quercetin as a cardiovascular agent. Eur. J. Med. Chem..

[3] Singh P., Arif Y., Bajguz A., Hayat S. (2021). The role of quercetin in plants. Plant Physiol. Biochem..

[4] Zazeri G., Ribeiro Povinelli A.P., Pavan N.M., de Carvalho D.R., Cardoso C.L., Ximenes V.F. (2021). Experimental studies and computational modeling on cytochrome c reduction by quercetin: The role of oxidability and binding affinity. J. Mol. Struct..

[5] Shabbir U., Rubab M., Daliri E.B.M., Chelliah R., Javed A., Oh D.H. (2021). Curcumin, Quercetin, Catechins and Metabolic Diseases: The Role of Gut Microbiota. Nutrients.

[6] Rauf A., Imran M., Khan I.A., Mujeeb U.R., Gilani S.A., Mehmood Z., Mubarak M.S. (2018). Anticancer potential of quercetin: A comprehensive review. Phytother. Res..

[7] Almeida A.F., Borge G.I.A., Piskula M., Tudose A., Tudoreanu L., Valentova K., Williamson G., Santos C.N. (2018). Bioavailability of Quercetin in Humans with a Focus on Interindividual Variation. Compr. Rev. Food Sci..

[8] Chikara S., Nagaprashantha L.D., Singhal J., Horne D., Awasthi S., Singhal S.S. (2018). Oxidative stress and dietary phytochemicals: Role in cancer chemoprevention and treatment. Cancer Lett..

[9] Zhao Y., Li Z.F., Zhang D., Wang Z.Y., Wang L. (2021). Quercetin alleviates Cadmium-induced autophagy inhibition via TFEB-dependent lysosomal restoration in primary proximal tubular cells. Ecotoxicol. Environ. Saf..

[10] Wu D., Chen Z. (2014). ZnS quantum dots-based fluorescence spectroscopic technique for the detection of quercetin. Luminescence.

[11] Ravichandran R., Rajendran M., Devapiriam D. (2014). Antioxidant study of quercetin and their metal complex and determination of stability constant by spectrophotometry method. Food Chem..

[12] Jing R.J., Jiang X.Y., Hou S.R., Li X.J., Yuan Z.B. (2007). Determination of quercetin, luteolin, kaempferol and isoquercitrin in stamen nelumbinis by capillary zone electrophoresis-ultraviolet detection. Chin. J. Anal. Chem..

[13] Zhang Q.L., Cui H. (2005). Simultaneous determination of quercetin, kaempferol, and isorhamnetin in phytopharmaceuticals of Hippophae rhamnoides L. by high-performance liquid chromatography with chemiluminescence detection. J. Sep. Sci..

[14] Jin D., Hakamata H., Takahashi K., Kotani A., Kusu F. (2004). Determination of quercetin in human plasma after ingestion of commercial canned green tea by semi-micro HPLC with electrochemical detection. Biomed. Chromatogr..

[15] Wang Q., Xue Q., Chen T., Li J., Liu Y., Shan X., Liu F., Jia J. (2021). Recent advances in electrochemical sensors for antibiotics and their applications. Chin. Chem. Lett..

[16] Kaya H.O., Cetin A.E., Azimzadeh M., Topkaya S.N. (2021). Pathogen detection with electrochemical biosensors: Advantages, challenges and future perspectives. J. Electroanal. Chem..

[17] Senocak A., Koksoy B., Akyuz D., Koca A., Klyamer D., Basova T., Demirbas E., Durmus M. (2019). Highly selective and ultra-sensitive electrochemical sensor behavior of 3D SWCNT-BODIPY hybrid material for eserine detection. Biosens. Bioelectron..

[18] Gutierrez F., Ortega G., Luis Cabrera J., Rubianes M.D., Rivas G.A. (2010). Quantification of Quercetin Using Glassy Carbon Electrodes Modified with Multiwalled Carbon Nanotubes Dispersed in Polyethylenimine and Polyacrylic Acid. Electroanalysis.

[19] Arvand M., Chaibakhsh N., Daneshvar S. (2015). Amperometric Determination of Quercetin in Some Foods by Magnetic Core/Shell Fe_3_O_4_@ZnO Nanoparticles Modified Glassy Carbon Electrode. Food Anal. Methods.

[20] Ma J.J., Yu Y.G., Yin S.W., Tang C.H., Yang X.Q. (2018). Cellular Uptake and Intracellular Antioxidant Activity of Zein/Chitosan Nanoparticles Incorporated with Quercetin. J. Agric. Food Chem..

[21] Han G.C., Li H., Ferranco A., Tao Z., Cheng Y., Chen Z., Xue M., Feng X.Z., Kraatz H.B. (2020). The construction of a simple sensor for the simultaneous detection of nitrite and thiosulfate by heme catalysis. RSC Adv..

[22] Han G.C., Hou J.T., Huang Z.L., Feng X.Z., Chen Z.C., Xiao W.X., Li S. (2017). Electrochemical Behaviors of Ferrocene Dicarboxylate and its Application for Heme Detection. Int. J. Electrochem. Sci..

[23] Han G.C., Feng X.Z., Liang J.T., Xiao W.X., Chen Z.C. (2016). Interaction Study of Ferrocene Derivatives and Heme by UV-Vis Spectroscopy. Spectrosc Spect Anal.

[24] Feng X.Z., Su X.R., Ferranco A., Chen Z.C., Han G.C., Jiang Z.L., Kraatz H.B. (2020). Real-Time Electrochemical Detection of Uric Acid, Dopamine and Ascorbic Acid by Heme Directly Modified Carbon Electrode. J. Biomed. Nanotechnol..

[25] Feng X.Z., Li H., Ferranco A., Chen Z., Xue M., Han G.C., Jiang Z., Kraatz H.-B. (2020). A Very Simple Method for Detection of Bisphenol A in Environmental Water by Heme Signal Amplification. J. Electrochem. Soc..

[26] Lee C.S., Kim T.H. (2021). Large-Scale Preparation of MoS_2_/Graphene Composites for Electrochemical Detection of Morin. ACS Appl. Nano Mater..

[27] Kathiravan D., Huang B.R., Saravanan A., Prasannan A., Hong P.D. (2019). Highly enhanced hydrogen sensing properties of sericin-induced exfoliated MoS_2_ nanosheets at room temperature. Sens. Actuators B Chem..

