# Supplementary material for: Convenient Heme Nanorod Modified Electrode for Quercetin Sensing by Two Common Electrochemical Methods
Source: Micromachines (Basel). 2021 Dec 7;12(12):1519. doi: 10.3390/mi12121519 (PMC8707963; doi:10.3390/mi12121519)
Supplement: Supplementary file 1 [file micromachines-12-01519-s001.zip › micromachines-1483725-supplementary.pdf]

Appendix B for

Convenient Heme Nanorod Modified Electrode for Quercetin Sensing by Two Common Electrochemical Methods

Jin-Guang Liu †, Jia-Zheng Wan †, Qing-Min Lin, Guo-Cheng Han*, Xiao-Zhen Feng* and Zhencheng Chen*

School of Life and Environmental Sciences, Guilin University of Electronic Technology, Guilin 541004, P. R. China; liujg1248798982@163.com (J.-G. L.); a1197797116@163.com (J.-Z. W.); lqm442049752@163.com (Q.-M. L.)

* Correspondence: hangc81@guet.edu.cn (G.-C. H.), fxz97118@guet.edu.cn (X.-Z. F.) and E-mail: chenzhch1965@163.com (C. Z. C.).

† These authors contributed equally to this work.

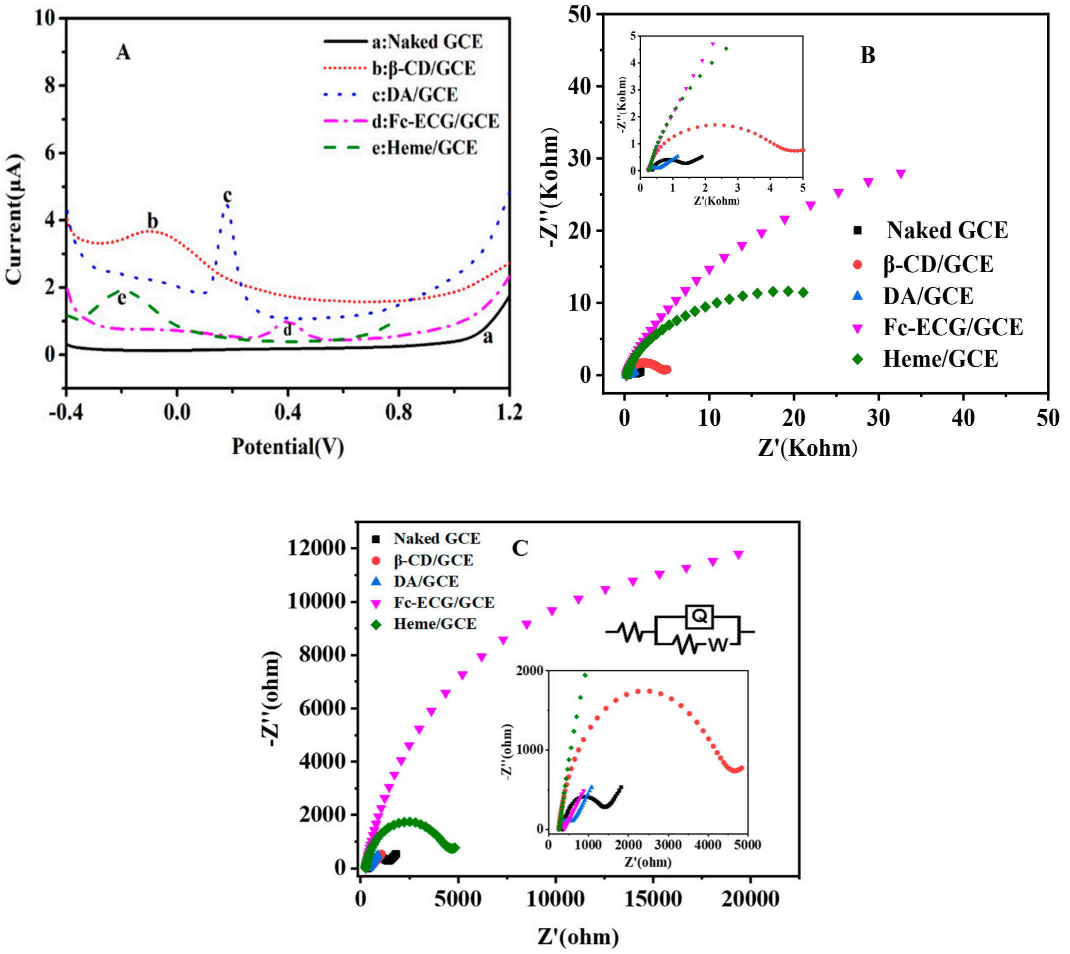

Figure S1: DPV graphs (A) of different working electrodes in PBS buffer (pH 7.0) and EIS diagrams (B) of different working electrodes in 5 mmol·L^{-1} $K_3[Fe(CN)_6]/K_4[Fe(CN)_6]$ solution; EIS fitting diagrams (C).

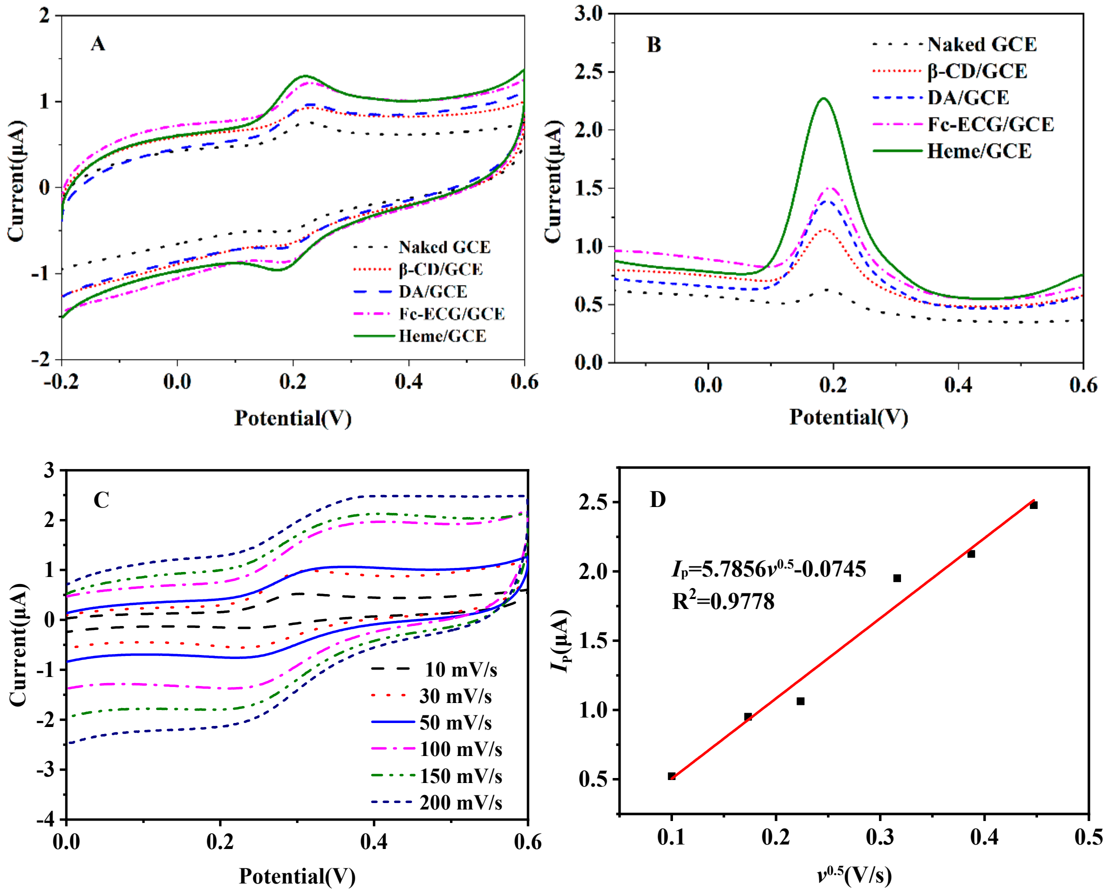

Figure S2: CV (A) and DPV (B) diagrams of 10 $\mu mol \cdot L^{-1}$ Qu on different working electrodes; (C) Cyclic voltammograms of Qu at Heme/GCE with different scan rate (10–200 mV/s); (D) The plots of anodic peak currents (Ip) and Qu *vs*. $v^{0.5}$.

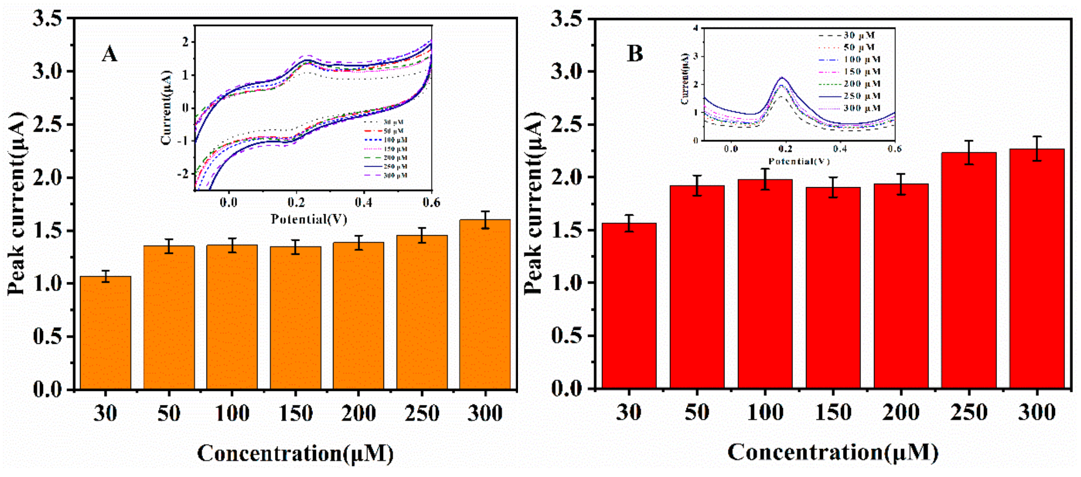

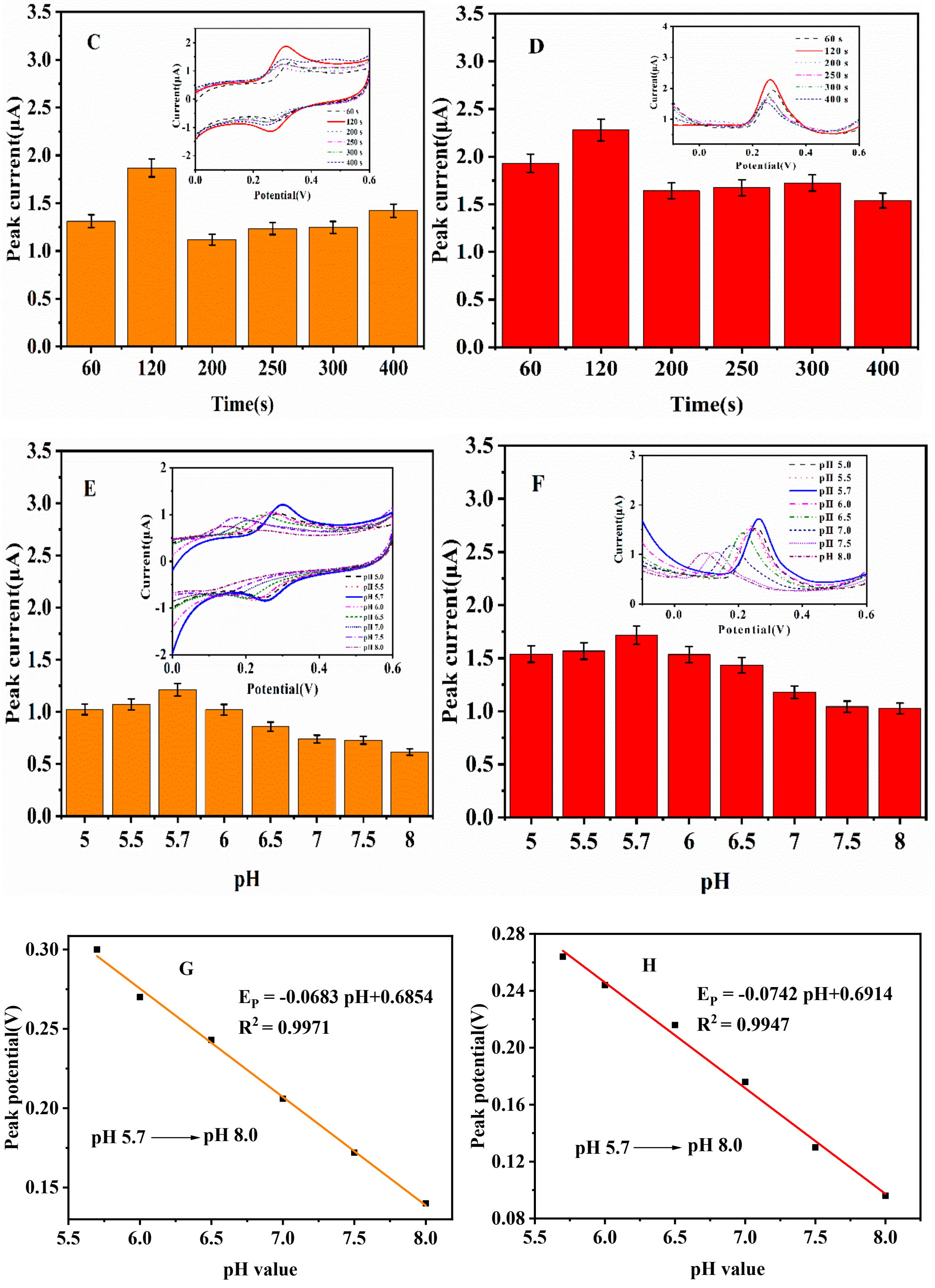
C
D
E
F
G
H
Peak current(μA)
Time(s)
pH
Potential(V)
Current(μA)
60 s
120 s
200 s
250 s
300 s
400 s
pH 5.0
pH 5.5
pH 5.7
pH 6.0
pH 6.5
pH 7.0
pH 7.5
pH 8.0
Peak potential(V)
pH value
E$_P$ = -0.0683 pH+0.6854
R^2 = 0.9971
E$_P$ = -0.0742 pH+0.6914
R^2 = 0.9947
pH 5.7 → pH 8.0

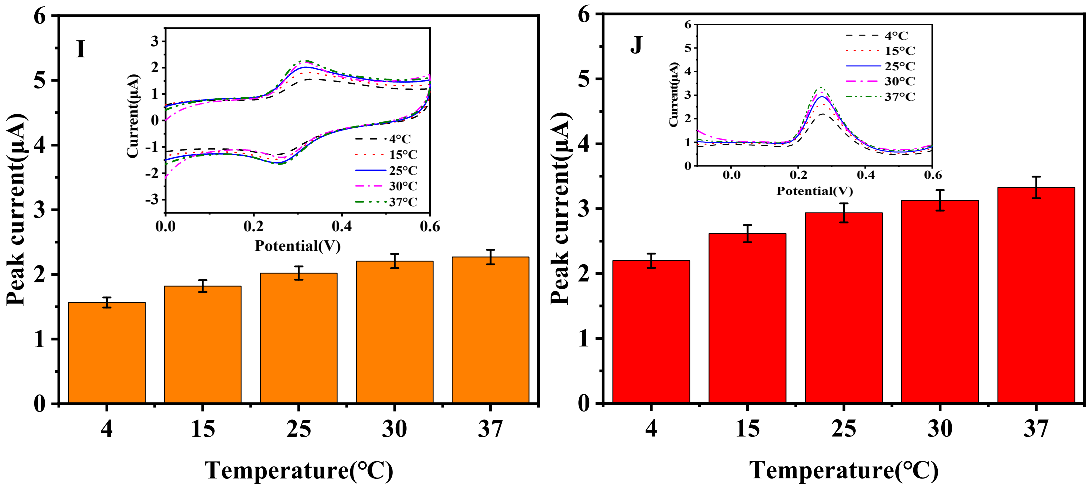

Figure S3: 10 μmol·L^{-1} Qu on working electrodes modified with different Heme concentrations (A) CV graph and (B) DPV graph; (C) CV graph and (D) DPV graph of the 10 μmol·L^{-1} Qu under different Heme deposition time; (E) CV graph and (F) DPV graph of the 10 μmol·L^{-1} Qu detected in PBS buffer with different pH; (G) The relationship between peak potential and pH value in CV method; (H) The relationship between peak potential and pH value in DPV method; (I) CV graph and (J) DPV graph of the 10 μmol·L^{-1} Qu at different temperatures.

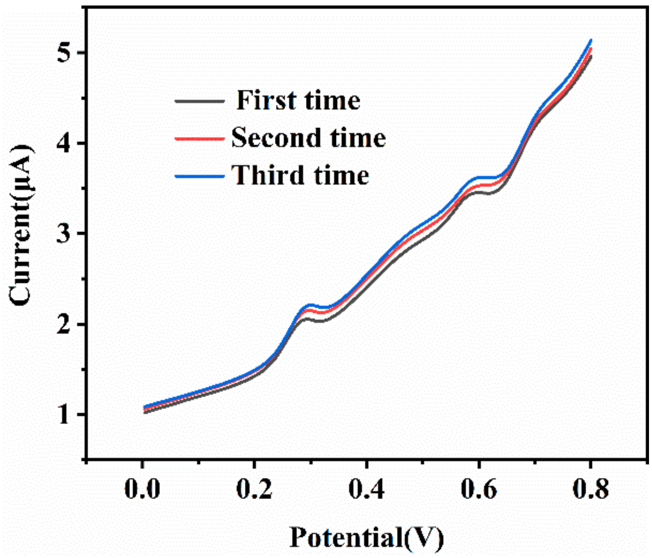

Figure S4: The DPV diagram of the actual sample tested three times in a row.

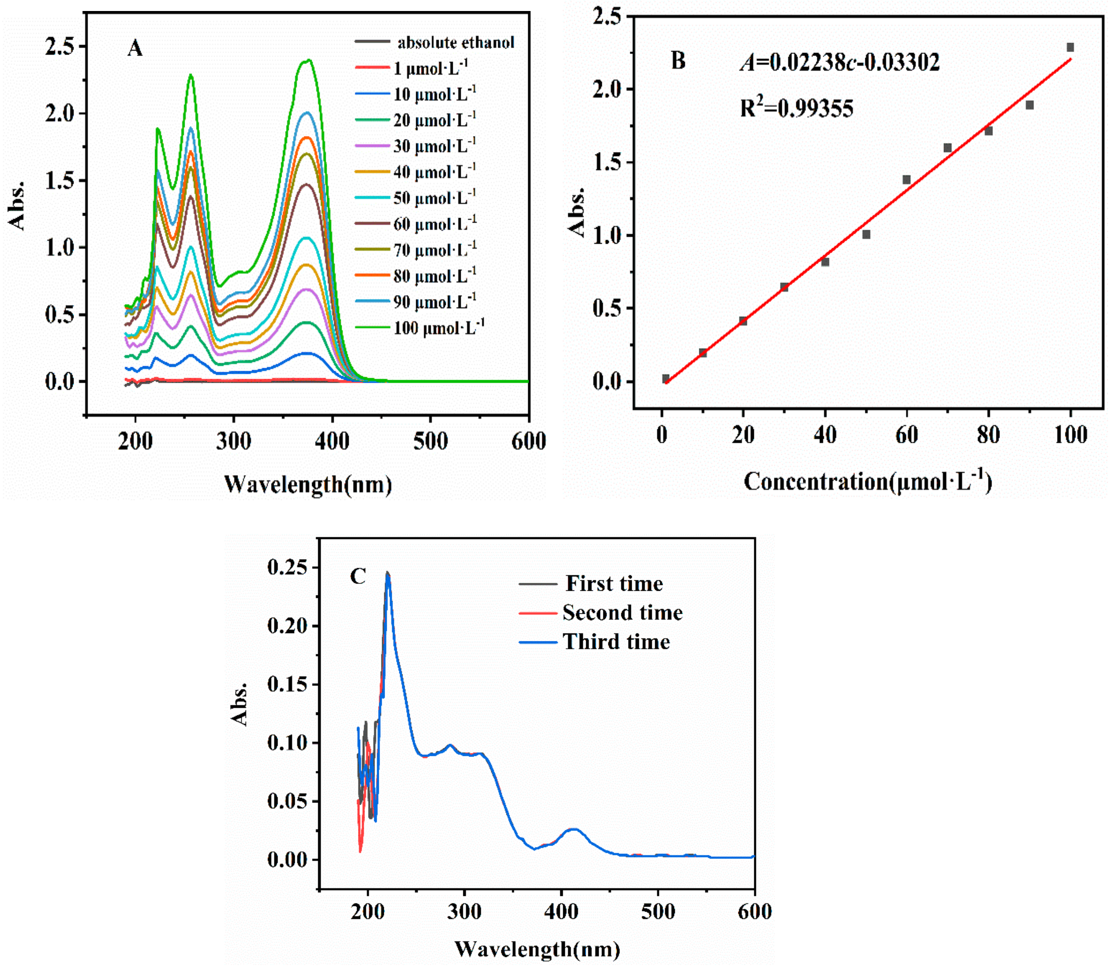

Figure S5: (A) UV-Vis spectra of different concentration of standard Qu solution, (B) the linear fitting curve of UV detection, and (C) UV-Vis spectra of actual samples were detected by three times.

Table S1: Comparison with other Qu detection methods

**Electrode**	**Detection method**	**Scope of test (μM)**	**Detection limit (μM)**	**References**
ZnO/CNS/MCPE/GCE	DPV	0.17-3.63	0.04	[1]
MIP/GO/GCE	CV	0.1-100	0.065	[2]
EDS/MCNTs/GCE	CV	0.995-47.6	0.036	[3]
Fe_3O_4@ZnO/CP/GCE	CV	0.79-61	0.16	[4]
Mn-doped/ZnS/QDs/GCE	DPV	0.33-20	0.16	[5]
$MnWO_4$/GCE	CV	16.7-74.4	-	[6]
WS_2/GCE	DPV	5.0-1000	1.20	[7]
MIP/MIL-101(Cr)/MoS_2 /GCE	DPV	0.1-700	0.02	[8]

Heme/GCE	DPV	0.1-700	0.063	this work
	CV	0.1-700	0.134	

References

1. Saritha, D.; Koirala, A. R.; Venu, M.; Reddy, G. D.; Reddy, A. V. B.; Sitaram, B.; Madhavi, G.; Aruna, K., A simple, highly sensitive and stable electrochemical sensor for the detection of quercetin in solution, onion and honey buckwheat using zinc oxide supported on carbon nanosheet (ZnO/CNS/MCPE) modified carbon paste electrode. *Electrochim. Acta* **2019**, *313*, 523-531. https://doi.org/10.1016/j.electacta.2019.04.188.
2. Sun, S.; Zhang, M.; Li, Y.; He, X., A molecularly imprinted polymer with incorporated graphene oxide for electrochemical determination of quercetin. *Sensors* **2013**, *13*, 5493-506. https://doi.org/10.3390/s130505493.
3. Zheng, Y.; Ye, L.; Yan, L.; Gao, Y., The Electrochemical Behavior and Determination of Quercetin in Choline Chloride/Urea Deep Eutectic Solvent Electrolyte Based on Abrasively Immobilized Multi-Wall Carbon Nanotubes Modified Electrode. *Int. J. Electrochem. Sci.* **2014**, *9*, 238-248. https://doi.org/10.1149/2.006402eel.
4. Arvand, M.; Chaibakhsh, N.; Daneshvar, S., Amperometric Determination of Quercetin in Some Foods by Magnetic Core/Shell Fe_3O_4@ZnO Nanoparticles Modified Glassy Carbon Electrode. *Food Anal. Methods* **2015**, *8*, 1911-1922. https://doi.org/10.1007/s12161-014-0080-8.
5. Zhang, Z.; Miao, Y.; Lian, L.; Yan, G., Detection of quercetin based on Al^{3+}-amplified phosphorescence signals of manganese-doped ZnS quantum dots. *Anal. Biochem.* **2015**, *489*, 17-24. https://doi.org/10.1016/j.ab.2015.08.002.
6. Muthamizh, S.; Suresh, R.; Giribabu, K.; Manigandan, R.; Kumar, S. P.; Munusamy, S.; Narayanan, V., MnWO4 nanocapsules: Synthesis, characterization and its electrochemical sensing property. *J. Alloys Compd.* **2015**, *619*, 601-609. https://doi.org/10.1016/j.jallcom.2014.09.049.
7. Durai, L.; Kong, C. Y.; Badhulika, S., One-step solvothermal synthesis of nanoflake-nanorod WS_2 hybrid for nonenzymatic detection of uric acid and quercetin in blood serum. *Mater. Sci. Eng. C-Mater.* **2020**, *107*, 110217. https://doi.org/10.1016/j.msec.2019.110217.
8. Kathiravan, D.; Huang, B.R.; Saravanan, A.; Prasannan, A.; Hong, P.D., Highly enhanced hydrogen sensing properties of sericin-induced exfoliated MoS_2 nanosheets at room temperature. *Sens. Actuators B Chem.* **2019**, *279*, 138-147. https://doi.org/10.1016/j.snb.2018.09.104.
